# A Mini Review of Ceramic-Based MOF Membranes for Water Treatment

**DOI:** 10.3390/membranes13090751

**Published:** 2023-08-24

**Authors:** Xueling Wang, Man Wang, Mingliang Chen, Yatao Zhang

**Affiliations:** 1 School of Chemical Engineering, Zhengzhou University, Zhengzhou 450001, China; xuelingwang@zzu.edu.cn (X.W.);; 2 Department of Water Management, Delft University of Technology, Stevinweg 1, 2628 CN Delft, The Netherlands

**Keywords:** ceramic membrane, metal–organic framework, water treatment, fabrication method

## Abstract

Ceramic membranes have been increasingly employed in water treatment owing to their merits such as high-stability, anti-oxidation, long lifespan and environmental friendliness. The application of ceramic membranes mainly focuses on microfiltration and ultrafiltration processes, and some precise separation can be achieved by introducing novel porous materials with superior selectivity. Recently, metal–organic frameworks (MOFs) have developed a wide spectrum of applications in the fields of the environment, energy, water treatment and gas separation due to the diversity and tunable advantages of metal clusters and organic ligands. Although the issue of water stability in MOF materials inhibits the development of MOF membranes in water treatment, researchers still overcome many obstacles to advance the application of MOF membranes in water treatment processes. To the best of our knowledge, there is still a lack of a reviews on the development process and prospects of ceramic-based MOF membranes for water treatment. Therefore, in this review, we mainly summarize the fabrication method for ceramic-based MOF membranes and their application in water treatment, such as water/salt separation, pollutant separation, heavy metal separation, etc. Following this, based on the high structural, thermal and chemical stability of ceramic substrates, and the high controllability of MOF materials, the superiority and insufficient use of ceramic-based MOF membranes in the field of water treatment are critically discussed.

## 1. Introduction

Membrane separation is one of the most important technologies used to address the issue of environmental water pollution. Compared with organic membranes, ceramic membranes have been increasingly employed in water treatment owing to their merits such as high-stability, anti-oxidation, long lifespan and environmental friendliness [1,2,3]. Currently, ceramic membranes are mainly used in microfiltration and ultrafiltration processes, and their application in precise separation is still in its infancy. Developing high-performance ceramic-based novel separation membranes is a key step to further expanding their application in precise separation, even in harsh conditions. Porous ceramic membranes are often used as substrates, and by introducing one or more separation layers with small pore sizes, they are expected to be used in various applications, including microfiltration, ultrafiltration, nanofiltration, reverse osmosis and gas separation [1].

**Figure 1:**
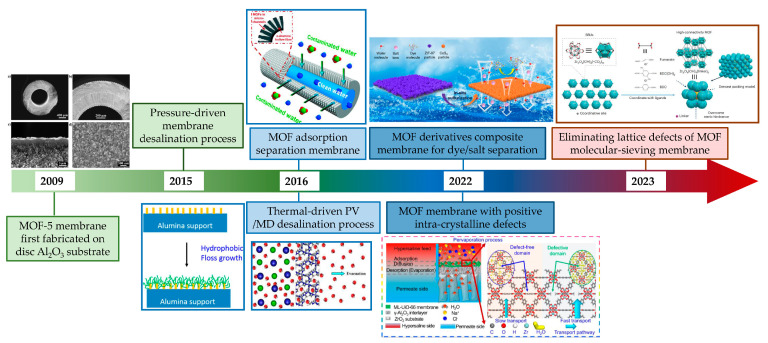
Development of ceramic-based MOF membrane for water treatment [13,14,15,16,17,18,19] (adopted with copyright permission from Elsevier and ACS publishers).

In recent years, the rapid development of nanoporous materials such as metal–organic frameworks (MOFs) has opened up a new avenue to fabricating nanoporous membranes on ceramic substrates. MOFs are one of the typical organic–inorganic hybrid materials with intricate pore structures, and are composed of metal ions and organic linkers. In addition, MOFs can be made with one-dimensional, two-dimensional or three-dimensional structures, making them a suitable building block for membrane fabrication. However, not all MOF materials can be employed in water treatment due to the poor water stability exhibited by many of them. Certain types of MOF materials that exhibit stability have demonstrated enhanced performance in wastewater treatment due to their structurally designable nature through pre- or post-modification [4]. Due to their exceptional properties, MOFs hold great potential for membrane separation application [5]. Given the requisite solvents and solvothermal temperatures for the fabrication of pure MOF membranes, ceramic membranes stand out due to their remarkable structural stability in both solvent environments and high temperatures. This sets them apart from organic membranes. In addition, due to the high stability of ceramic membranes, the development of a high-stability MOF separation layer is expected to be realized for the precision separation of ceramic-based membranes in the harsh water treatment field. Therefore, combining the advantages of ceramic membranes and nanoporous MOF materials to construct a high-performance high-stability ceramic-based MOF membrane is of great significance for ceramic-based membranes in precision separation in water treatment. However, the primary applications of MOF membranes have been focused on gas separation rather than water treatment due to the instability of most MOF structures and the challenges associated with fabricating continuous dense and stable MOF membranes [6,7,8]. For ceramic-based MOF membranes, the MOF-5 membrane was first fabricated on an Al_2_O_3_ disc substrate. Following this, different water-stabilized MOF membranes and different membrane processes have been employed in water treatment. Recently, there have been some very valuable reports about improving membrane performance by regulating the intra-crystalline structure (Figure 1). There are already some reviews about the application of ceramic membranes or MOF membranes. Dong et al. summarized the ceramic membrane applications in different harsh environments, such as oil/water separation, saline water and emerging contaminant wastewater treatment [2]. Hu et al. presented recent advances in MOF membranes for water treatment [9]. Lai et al. summarized the MOF membranes used for gas separation and focused on the separation of various gases [10]. Very recently, Eddaoudi et al. discussed the latest advances in MOF-based membranes in detail [11]. Wang et al. systematically highlighted the research progress on substrates for the preparation of MOF membranes in separation [12]. However, there is still a lack of a comprehensive summary, including the development status, advantages and disadvantages of ceramic-based MOF membranes for water treatment. With this aim, this critical review presents an overview of the fabrication and water treatment application of ceramic-based MOF membranes, while also discussing the advantages and prospects of using ceramic-based MOF membranes for water treatment.

## 2. Fabrication of Ceramic-Based MOF Membrane

As a typical porous crystal material, the fabrication methods for MOF membranes are the same as for other zeolite membranes or molecular sieve membranes. Their high density, high crystallinity, high binding force between the crystal and substrate, thin membrane, etc., are the greatest concerns in the research on MOF membranes, and new substrates and new methods are being explored gradually [20]. To the best of our knowledge, there are three main types of water-stable MOFs: metal azolate frameworks, metal carboxylate frameworks with high-valence metals and some special functional MOFs. Among them, UiO-66 series MOFs (a typical metal carboxylate framework) and ZIFs series MOFs (a typical metal azolate framework) are usually fabricated via solvothermal methods, mechanochemical methods, microwave-assisted methods, electrochemistry methods and other methods [4,21]. The main traditional preparation methods for MOF membranes are as follows.

1. In situ growth method

In situ growth is the most direct and simple method for preparing MOF membranes, and involves putting one or more substrate into a mother solution for a hydrothermal or solvothermal reaction. There are two main stages: the nucleation stage and growth stage. During nucleation, the crystal grows randomly and adheres to the surface of the substrate or the pore. During the growth period, the crystals grow continuously around the nucleus, crosslinking and undergoing misgeneration, and a continuous membrane layer is finally formed.

**Figure 2:**
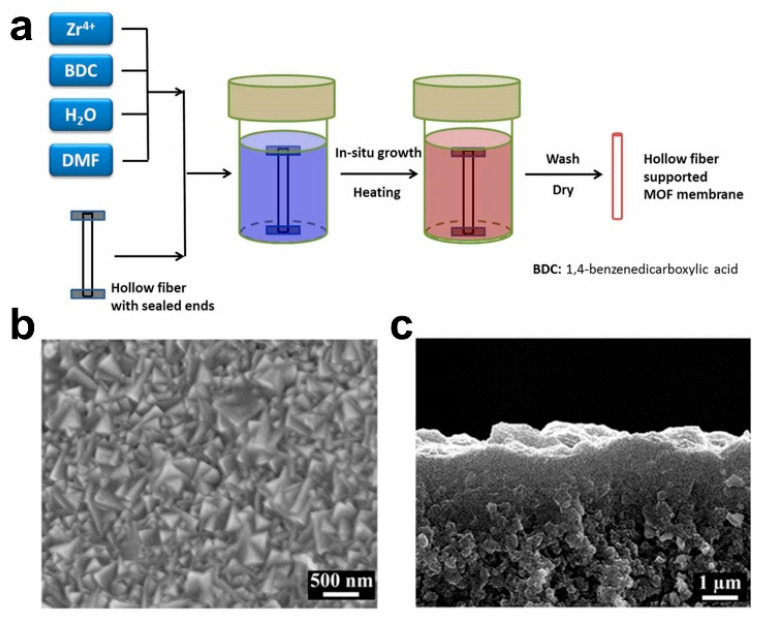
(**a**) Schematic diagram, (**b**) surface scanning electron microscope (SEM) image and (**c**) cross-sectional SEM image for one of typical UiO-66 membrane fabricated via in situ solvothermal method [14].

In 2005, Roland et al. first fabricated a MOF-5 membrane via a traditional in situ method (solvothermal or hydrothermal synthesis) successfully [22]. Research on the fabrication and application of MOF membranes has shown rapid development since the MOF separation membrane (i.e., the MOF-5 membrane fabricated via an in situ method [13]) was reported in 2009. Subsequently, compared with only some crystals scattered on the substrate surface, Zhu et al. prepared an oriented MIL-53 membrane with a thickness of 3 μm on an activated Al sheet [23]. In 2013, Jeong et al. synthesized a ZIF-8 membrane via an in situ method [24]. Recently, we fabricated an ultrathin missing-linker UiO-66 membrane of 103 ± 14 nm on an γ-Al_2_O_3_-modified ZrO_2_ substrate via an in situ method [18]. In short, MOF membranes have been fabricated successfully via in situ methods, including the UiO-66 membrane (Figure 2) [14,25,26], ZIF-8 membrane, MIL-53 membrane [27], etc. The constraining factors of fabricating high-quality MOF membranes are their substrate properties, the composition of the synthesized mother solution and the energy supply mode. The significant advantages of the in situ method are the simple operation conditions, which are favorable for realizing industrial application. However, the synthesis conditions are relatively strict, and the membrane quality is affected by various factors, resulting in the formation of some separate crystals and a thick membrane. As the nucleation rate of crystals on the substrate surface is greatly affected by the chemical properties of the substrate, usually, the nucleation and growth of MOF crystals occur preferentially in solution, rather than on the substrate surface. Therefore, MOFs often have a low growth rate on various support surfaces, which leads to great difficulties in the synthesis of MOF membranes using in situ growth method [28]. The physicochemical properties of the substrate surface play a key role in the fabrication of high-quality MOF membranes via in situ growth methods. Therefore, it is necessary to modify the ceramic substrates to promote the well-growth ability for MOF crystals on the substrate surface before employing an in situ method to synthesize MOF membranes.

The in situ method is favorable for fabricating a dense and continuous MOF membrane via a simple process, but the quality and thickness of the MOF membrane layer is hard to control due to the strict demand of the substrate surface properties and the long synthesis time. Not all MOF membranes can be grown effectively via in situ methods due to the homogenous nucleation and grown in the precursor solution. Functionalization of the ceramic substrate is often a necessary procedure before using an in situ growth method.

2. Secondary growth method

**Figure 3:**
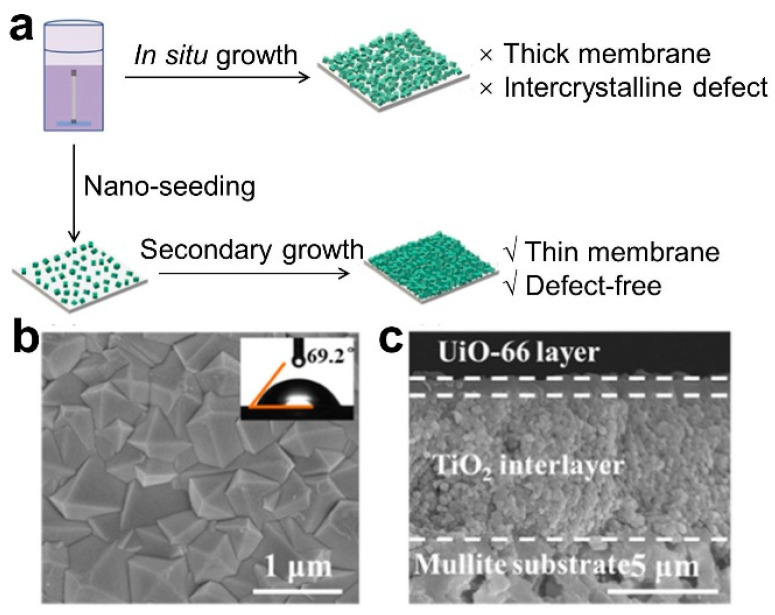
(**a**) Schematic diagram, (**b**) surface SEM and (**c**) cross-sectional SEM image for UiO-66 membrane fabricated via secondary growth method [31].

The secondary growth method, also known as the seeding method, is a common method for preparing thin membranes. In 1994, Lai et al. first proposed the concept of secondary seeding growth to prepare zeolite molecular sieve membranes [29]. Owing to the low heterogeneous nucleation density of MOF materials on traditional ceramic substrates, the seeding method has gained significant attention for the preparation of MOF membranes. There are two main steps in this method: (1) The MOF seed layer is introduced; this is the initial step involved the implantation of nano-MOF seeds onto the membrane substrate using methods such as coating, deposition, and others, all carried out at room temperature. This results in the formation of a loosely bound seed layer, primarily due to physical adhesion or weak chemical bonding. Simultaneously, techniques such as microwave treatment, thermal heating, or functionalization with metal compounds/organic materials are employed to enhance the adhesion of MOF seeding onto the ceramic substrate. (2) Following this, a secondary growth process is executed on the seeded substrate to establish the membrane layer, ultimately yielding a continuous MOF membrane (Figure 3). Li et al. fabricated a continuous and dense ZIF-7 membrane using the seeding method [30]. Dong et al. reported a UiO-66 nano-seeding layer grown on a TiO_2_-modified mullite substrate via an in situ method, and then, the thickness of the UiO-66 membrane grown through secondary growth was reduced to approximately ~1 μm [31].

One advantage of the secondary growth method is that it allows for the precise regulation of crystal growth, membrane thickness and microstructure, thanks to the partition of nucleation and growth periods, which minimizes the synthesis time and inhibits hetero crystal growth. This method is ideal for fabricating defect-free dense membranes. Nevertheless, the method needs high-quality crystal seeds, often of nano-scale size, to avoid the formation of large inter-crystalline defects during secondary growth. Additionally, the seeds must be distributed evenly in the seed solution, and the selectivity of MOF crystals is severely limited by the challenge of preparing uniform nanoscale crystal seeds and the exacting requirements for their good solvent dispersion. Hence, a key challenge in the secondary seed growth method is to generate small, uniform, high-quality seeds, and ensure that they can coat the substrate membrane surface evenly.

3. Layer-by-layer method

**Figure 4:**
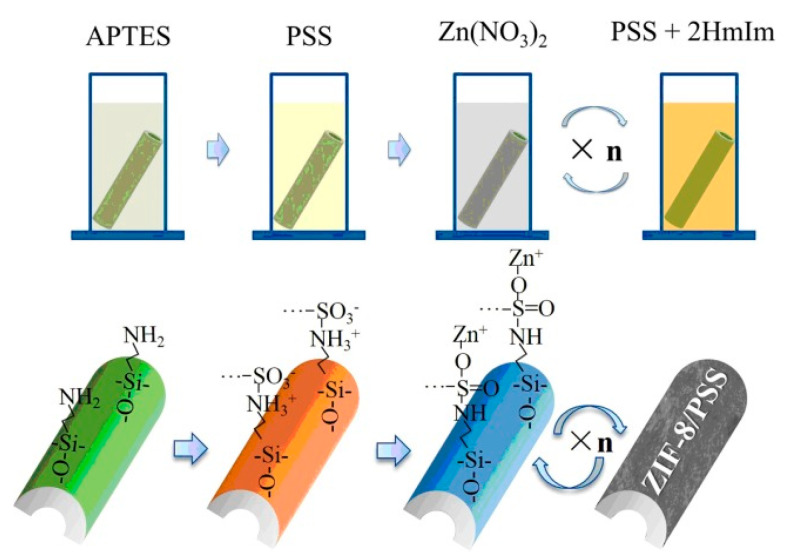
Schematic diagram of ZIF-8/PSS membrane fabricated on tubular Al_2_O_3_ substrate via layer-by-layer growth method [32].

The layer-by-layer method involves immersing the modified substrate into both a metal solution and an organic ligand solution for some time (Figure 4) [32]. Shekhah et al. [33] first utilized this approach to prepare a multilayer-oriented Zn(II)-BTC membrane, and briefly described the preparation method and phenomenon. Then, Shekhah et al. used this method to synthesize a Cu_2_(BTC)_3_ membrane again [34], and the membrane structure and performance were studied systematically. In 2013, Wiederrecht et al. [35] also synthesized DA-MOF and L2MOF porphyrin membranes via the layer-by-layer growth method. Li et al. [32] prepared ceramic-based Zn-MOF membranes using this method to remove dye molecules from water in the nanofiltration process. One advantage of this method is that the fabrication process can be carried out at room temperature, effectively decreasing the synthesis temperature. Owing to the separate steps, the precursor concentration and the cycle number can be controlled precisely; thus, the membrane thickness is controllable. Also, this fabrication method could be employed to achieve an oriented MOF membrane.

4. Microwave-induced thermal deposition method

**Figure 5:**
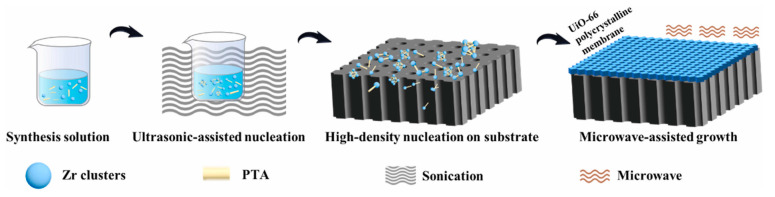
Schematic diagram of polycrystalline UiO-66 membrane fabricated via the microwave-assisted growth method [37].

Microwave-induced thermal deposition (MITD) is another simple fabrication method using microwave heating instead of the traditional solvothermal heating mode for MOF membranes. Jeong et al. fabricated a thin MOF-5 membrane via microwave-assisted technology [36]. Using the microwave-assisted method, Wei et al. fabricated ultrathin 210 nm UiO-66 membranes in only 1 h, which reduced the synthesis time significantly (Figure 5) [37]. In this method, a local high temperature was formed near the substrate surface, which is a necessary condition for MOF synthesis. Thus, the MOF crystallization phenomenon is more inclined to appear near the thin crystal plane near the substrate surface. The thermal conductivity of the substrate is a key factor in this method, and the superior thermal conductivity properties of the substrate are favored for improving MOF nucleation and membrane growth. Graphite, amorphous carbon and metal like Au/Pd possess good thermal conductivity; thus, they are excellent selected substrates for MOF membranes. The advantage of this method is that it can accelerate the MOF nucleation rate and reduce the reaction time.

5. Other methods

Along with the development of MOF materials, some other new methods for the fabrication MOF membranes are appearing gradually. Guo et al. prepared a continuous dense Cu_3_(BTC)_2_ membrane on copper nets via the “Double copper source method”, whereby the copper net could provide homogeneous nucleation that was favorable for obtaining a uniform and smooth membrane layer [38]. Jeong et al. fabricated an effectively growing MOF membrane via rapid heating deposition (RTD) in a very short time (only about ~10 min) successfully [39]. Brown et al. synthesized a high-quality ZIF-8 hollow fiber membrane via the reverse diffusion microflow technique [40]. Preparing MOF membranes through in situ growth methods is a challenging task. As a result, substrate modifications are often necessary to facilitate the growth of MOF crystals on the substrate surface. Among the various approaches, secondary seeding growth has emerged as a popular method for MOF membrane preparation due to its numerous advantages. In addition, the chemical bonding in the modified substrate can increase the bonding force between the membrane layer and substrate, so it has a great development potential.

Although there are several technologies available for preparing MOF membranes (Table 1), the thicknesses of MOF membranes fabricated via traditional in situ growth or secondary growth, such as the ZIF and UiO-66 families [14,41], often range from a few to tens of microns. Furthermore, these membranes also follow the “trade-off” effect between permeability and selectivity. This “trade-off” effect can be overcome by developing ultrathin membranes (≤1 μm), which have the potential for efficient separation. In recent years, a series of strategies have been developed for the preparation of ultrathin membranes, such as the layer-by-layer assembly method [42], reverse diffusion method [43], vapor deposition method [44], gel vapor deposition (GVD) method [45], self-assembly of 2D nanosheets [46,47], etc. These methods offer an attractive approach to preparing ultrathin membranes that possess high permeability due to their significantly reduced transmission resistance. Although the preparation method of ultrathin membranes has great prospects, a key technical bottleneck still remains in the scaling-up of its application due to its complex process, time-consuming nature and high cost.

Overall, the quality of a MOF membrane mainly depends on the characteristics of the substrate, the composition of the mother solution, the synthesis temperature and time, etc. Among these, the properties of the ceramic substrate are crucial for the fabrication of high-quality MOF membranes. Rigorous ceramic substrates with a smooth surface and small pore size are usually required in in situ growth processes. Pre-seeding and secondary growth are frequently employed for the growth of MOF membranes, especially when using ceramic substrates with macro-porous and rough surfaces. It is difficult to obtain dense MOF membranes on ceramic substrates with a thickness of <100 nm, which is limited by the grain size and the effective growth effect of 3D MOF crystal. Ultrathin MOF membranes with a thickness of <100 nm are usually 2D MOF membranes, which are mainly fabricated via drop coating, spin coating, etc. For the preparation of water-stable MOF membranes on ceramic substrate, comprehensive evaluation is necessary, including of the separation obstacle, the type and properties of the ceramic substrate, the structural characteristics of the water-stable nanoporous MOF, feasible fabrication methods, etc.

## 3. Water Treatment Application

Despite the poor stability of MOF structures in water, some researchers can still overcome various obstacles and promote the research progress of MOF membranes in liquid separation (mainly water treatment) efficiently (Table 2) [6,7,8]. Different from the transport of gas molecules, the diffusion resistance of liquid water molecules in MOF membranes is mainly due to the collision between water molecules, and the transport process can be described by the dissolution (adsorption)–diffusion mechanism [15]. Currently, the application of MOF membranes in water treatment mainly focuses on pressure-driven filtration desalination, pervaporation desalination, ion separation, pollutant separation and other fields (Table 2).

### 3.1. Water/Salt Separation

**Figure 6:**
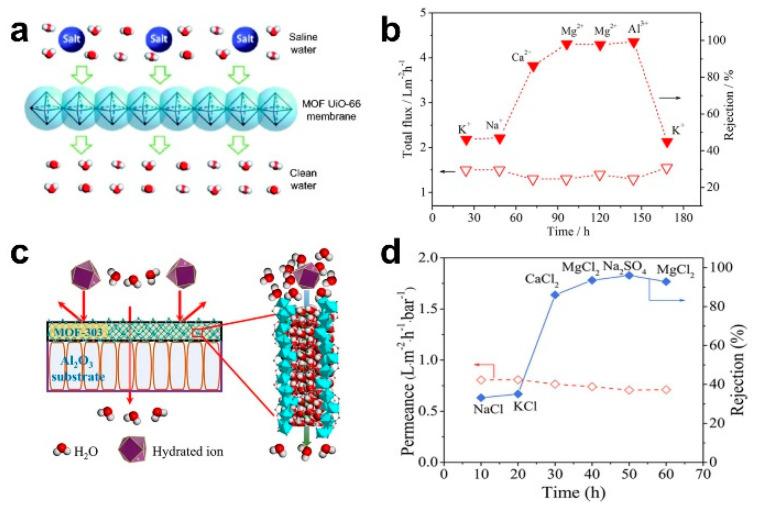
(**a**) Schematic diagram and (**b**) desalination performance of UiO-66 membrane under pressure-driven filtration [14]; (**c**) schematic diagram; and (**d**) desalination performance of MOF-303 membrane under pressure-driven filtration [52].

The pressure-driven desalination of ceramic-based MOF membranes can enable the efficient rejection of bivalent and trivalent ions. Liu et al. prepared a MOF UiO-66 membrane on an Al_2_O_3_ hollow fiber ceramic substrate via an in situ method. Based on the molecular sieving mechanism, the rejection rates of Ca^2+^, Mg^2+^ and Al^3+^ were 86.3%, 98.0% and 99.3%, respectively (Figure 3a,b) [14]. Cong et al. also prepared a MOF-303 membrane on an Al_2_O_3_ ceramic substrate via an in situ solvothermal method. Based on the mechanism of molecular sieving and electrostatic exclusion, the rejection rates of MgCl_2_ and Na_2_SO_4_ were up to 93.5% and 96.0%, respectively (Figure 6c,d) [52].

**Table 2:** List of ceramic-based MOF membranes for water treatment.

No.	Membrane Type	Substrate	Fabrication Method	Thickness	Application	References
1	UiO-66	Al_2_O_3_ hollow fiber	In situ solvothermal	~2 μm	Desalination	[14]
2	UiO-66	Al_2_O_3_ hollow fiber	Secondary growth and post modification	3.5 μm	Desalination	[53]
3	UiO-66	TiO_2_-modified Al_2_O_3_	Secondary growth	1 μm	Desalination	[29]
4	MOF-303	Al_2_O_3_ Disc	In situ solvothermal	4 μm	Desalination	[52]
5	UiO-66-NH_2_	APTES-modified Al_2_O_3_	Secondary growth	1 μm	Desalination	[54]
6	UiO-66	Al_2_O_3_	In situ solvothermal	--	Fluorine removal	[55]
7	UiO-66-NH_2_	AAO	In situ reaction cell	<500 nm	Ion separation	[56]
8	UiO-66	Al_2_O_3_	Vacuum filtration	--	Arsenic removal	[16]
9	UiO-66-NH_2_	ZrO_2_	Microwave-assisted	--	Plumbum removal	[57]
10	UiO-66	Al_2_O_3_	In situ solvothermal	--	Humic acid removal	[58]
11	ZIF-8	PDA-modified Al_2_O_3_	In situ solvothermal	20 μm	Desalination	[15]
12	CAU-1	Al_2_O_3_	Secondary growth	1.3 μm	Desalination	[59]
13	NH_2_-MIL-53(Al)	Al_2_O_3_	Secondary growth	--	Desalination	[27]
14	ZIF-8	AAO	GO interface-assisted and secondary growth	0.45 μm	Ion separation	[60]
15	ZIF-300	Al_2_O_3_	Secondary growth	10 μm	Heavy metal and dye removal	[61]
16	ZIF-L	Al_2_O_3_	Secondary growth	6 μm	Anti-bacterial	[62]
17	MIL-53	AAO	In situ solvothermal	600 nm	Ion transport	[63]
18	2D Zn-MOF crosslinked nanosheet	Al_2_O_3_	Vacuum filtration	--	Dye removal	[64]
19	Al-MOFnanosheet	AAO	Vacuum filtration	20 nm	Desalination	[65]
20	ZIF-8/PSS	Al_2_O_3_	Layer-by-layer	5 μm	Dye removal	[32]
21	SSP@ZIF-8	AAO	In situ embedding	500 nm	Ion separation	[66]

Different from pressure-driven filtration, through which is difficult to achieve the efficient rejection of monovalent salt ions, a ceramic-based MOF pervaporation membrane can effectively overcome this problem. Zhu et al. prepared ZIF-8 membranes on a dopamine-modified Al_2_O_3_ ceramic membrane via an in situ solvothermal method, and the seawater desalination performance of the ZIF-8 membranes was verified using experiments and simulation calculation [15]. Meanwhile, the ZIF membranes showed excellent long-term stability in seawater. At 25, 50, 75 and 100 °C, the water fluxes of the ZIF-8 membranes were 5.8, 8.1, 10.8 and 13.5 L m^−2^ h^−1^, respectively, and the ion rejection was as high as 99.8%. It is expected that ZIF membranes can be applied in desalination. Wan et al. prepared UiO-66-NH_2_ membranes on 3-aminopropyltriethoxy-silane-modified Al_2_O_3_ ceramic membranes via an in situ solvothermal method, which were used for seawater desalination through a pervaporation process [54]. UiO-66-NH_2_ membranes exhibited high desalting performance, mainly due to the narrow pore size between water molecules and hydrated ions. When the temperature of the raw material increased from 45 to 90 °C, the water flux increased from 1.5 to 12.1 L m^−2^ h^−1^, and the ion rejection was maintained at 99.7%. Dong et al. prepared a UiO-66 membrane on a TiO_2_-modified mullite substrate. A high water flux (37.4 L m^−2^ h^−1^) was obtained when treating brine with a concentration of 7.0 wt.% at 85 °C. It also showed excellent operating stability in harsh environments (acidic, high-temperature and hypersaline water) [31]. We also constructed a robust ultrathin sub-nanopore ML-UiO-66 membrane layer on γ-Al_2_O_3_ interlayer-modified coarse ceramic substrates via a substrate surface engineering protocol combined with an in situ growth method [18]. The nanoporous γ-Al_2_O_3_ interlayer provided more heterogeneous nucleation sites and lowered roughness, favoring the growth of ultrathin well-inter-grown ML-UiO-66 membranes (103 ± 14 nm). Ultrathin ML-UiO-66 membranes are demonstrated to have not only excellent stability in harsh environments but also almost complete salt rejection, and more importantly, high water flux (~29.8 L m^−2^ h^−1^), outperforming existing state-of-the-art zeolite and MOF membranes [11]. Wanqin Jin et al. reported that in ceramic-based Zr-MOF membranes, by varying their ligand/secondary building unit (SBU) stoichiometric ratios by factors of 1.5 to 20, the water/salt selectivity of the optimized Zr-MOF membranes can be as high as 9000 [19].

Forword osmosis (FO), which is the only membrane process with an osmotic pressure gradient as a main driving force, has also been used for ceramic-based MOF membranes for desalination application. MIL-140B membrane growth on an Al_2_O_3_/YSZ substrate had a water flux of 12.023 L m^−2^ h^−1^ and reverse solute flux of 0.094 L m^−2^ h^−1^, better than other mixed-matrix membranes, thin film nanocomposite (TFN) membranes and other FO membranes [67].

Therefore, for water/salt separation via a pressure-driven filtration process, ceramic-based MOF membranes present superior selectivity for multivalent salt ions owing to the synthetic effect of molecular sieving and electrostatic exclusion, but low rejection for monovalent salt ions due to the dehydration effect when water molecules pass through the MOF nanopore channel. Fortunately, ceramic-based MOF membranes can effectively reject monovalent salt ions via thermally driven pervaporation or the osmotically driven FO membrane process. Therefore, the effective separation of water/salt with different valences could be addressed using ceramic-based MOF membranes with an appropriate membrane process.

### 3.2. Pollutant Separation

Environmental pollutants, such as dye, micro-pollutants, fluoride, etc., lead to severe water pollutions issues. Among the different treatment technologies, the membrane process can offer a very wide spectrum of pollutant separation, and its performance also follows the trade-off effect between permeability and selectivity. To overcome the difficulty of traditional membrane separation for target molecules through size exclusion. Adsorption membranes provide an easy and convenient method. Jinhuai Liu et al. [55] fabricated an Al_2_O_3_-based Zr-MOF adsorbent membrane for rapid fluoride removal via dynamic filtration, and the effect of initial fluoride concentration and flow rate on fluoride removal efficiency was studied. Utilizing a Zr-MOF adsorbent membrane with a thickness of 20 μm, the fluoride removal capacity achieved an impressive 5510 L m^−3^ when treating a fluoride solution with an initial concentration of 5 mg L^−1^. Characterization via XPS and FT-IR revealed that the surface hydroxyl groups and metal active sites dominated the fluoride adsorption mechanism. Additionally, MOF or MOF-derived materials were also employed as additives to enhance the pollutant removal efficiency. Xiao Hu et al. fabricated a robust ceramic-based PA layer with Al-based MOF MIL-53 as the connected interlayer for nanofiltration with high rejection (>99%) for a 100 mg L^−1^ RhB solution; this method can overcome the challenge of preparing an organic layer on an inorganic substrate [68]. MOFs play an important role in enhancing the physical connection between the ceramic substrate and the polyamide separation layer, thus effectively improving the nanofiltration performance of the membrane. Naixin Wang et al. fabricated a ZIF-67-derived CoSx composite membrane through the synergistic combination of a solvothermal method and an in situ sulfurization process, aiming for effective dye/salt separation. The composite membranes possessed high water permeance (751.6 LMH/MPa), high rejection (>99.5%) for different drug dye molecules, such as TC (MW = 444) and CYC (MW = 885), and low salt rejection (<18.7%) [17]. However, when dealing with small-molecular dyes such as MO (MW = 327), the membranes exhibited a rejection rate of only 63.7%. Thus, pore size sieving was considered a primary separation mechanism for membranes of this type, enabling efficient selectivity between dye molecules and salts. Owing to the high stability of ceramic membranes and the designability of the MOF structure, it has great potential for application in studying ceramic-based MOF membranes in pollutant removal.

### 3.3. Heavy Metal Separation

Heavy metals, such as lead (Pb), nickel (Ni), copper (Cu), zinc (Zn) and others, originated from various chemical industries, and are frequently found in industrial wastewater. These heavy metal ions will have a serious negative influence on human health and the ecological environment, owing to their circulation in the food chain and accumulation in organisms.

Owing to the superior chemical and thermal stability of ZIF materials, ceramic-based ZIF membranes have been employed in the removal of heavy metal ions. Wanqin Jin et al. reported on the thermal and chemical stability of ZIF-300 MOF membranes on an Al_2_O_3_ substrate for the efficient removal of heavy metal ions from wastewater. The ZIF-300 membranes exhibited water permeance of 39.2 L m^−2^ h^−1^ bar and a 99.21% rejection rate for CuSO_4_ [61]. The positron annihilation spectroscopy (PAS) technique was initiatively employed to characterize the membrane hierarchical microstructure. As the synthesis temperature increased from 80 to 120 °C, the membrane’s pore size decreased from 3.553 to 3.117 Å. This decrease can be attributed to the fact that higher temperatures are favorable for the effective growth of MOF crystals. The enhanced rejection of metal ions and dyes was mainly attributed to the size exclusion mechanism. Mugahed Amran et al. reported on a ZIF-8 adsorption membrane supported on an Al_2_O_3_ substrate, with an adsorption capacity of 115.38 mg g^−1^, leading to an average lead (II) removal rate up to 92.13%. Also, the membrane showed high water permeation of 288.41 L m^−2^ h^−1^ bar^−1^ [69]. By fitting kinetic and isotherm models to the experimental data, it was determined that chemisorption is the primary adsorption mechanism [70].

At present, compared with the current research progress on membrane separation for the removal of heavy metal ions, studies on ceramic-based MOF membranes applied in the field of heavy metal removal urgently need to be strengthened. Some real heavy metal ion separation in harsh water environment systems, such as acid/alkaline environments, could be conducted using ceramic-based water-stable MOF membranes due to their superior long-term operation stability. Also, the complex mechanism between membrane structure and membrane performance should be emphasized.

Although MOF membranes have shown promise in various wastewater treatment applications, such as water/salt separation, pollutant removal and heavy metal separation, using different membrane processes (pressure-driven, thermally driven, osmotically driven), the focus should now shift toward achieving even more precise separations, particularly in challenging environmental conditions. This highlights the potential for ceramic-based MOF membranes to be harnessed effectively.

## 4. Conclusions and Prospects

Ceramic membranes have attracted much attention in water treatment areas, such as protein separation, organic wastewater treatment, etc. Some harsh and precise separation system problems could also be solved using ceramic membranes after introducing novel nano-material as a separation layer. MOFs have attracted extensive attention owing to their unique advantages. This review provides an overview of ceramic-based MOF membranes, including their fabrication methods and water treatment applications. The water treatment application of ceramic-based MOF membranes mainly focuses on 3D MOF membranes, such as water-stable MOF membranes (e.g., UiO-66, MOF-303), which showed superior desalinating ability. ZIF-67-derived CoSx composite membranes were used for dye/salt separation. ZIF-8 adsorption membranes on alumina were used for the removal of lead (II). The separation mechanism for water/salt separation is based on the molecular sieving or dehydration effect. The rejection mechanism for pollutants and heavy metal ions mainly involves adsorption or the size sieving effect.

Overall, although superior performance (high rejection, anti-acid/base ability, long-term operation stability) was demonstrated in treating hypersaline water, ceramic-based MOF membranes for water treatment are still in their infancy. The issues of low permeability and weak anti-oxidant ability urgently need to be solved. And long-term water stability is also a key issue that must be considered. Based on the designability of the MOF pore structure, understanding the relationship between the physiochemical properties of the MOF pore structure and its separation performance will favor the rational design of the next generation of ceramic-based MOF membranes for enhanced performance, paving a new avenue for the use of ceramic-based MOF membranes in the treatment of hypersaline water and other water treatment applications, such as ion separation, organic separation and actual separation in harsh environments. More importantly, the two key challenges of economics and environmental friendliness should be given high priority to drive the development of ceramic-based MOF membranes towards practical applications in the near future.

## Figures and Tables

**Table 1:** Comparison of mainstream fabrication methods for MOF membranes.

Fabrication Method	Advantages	Disadvantages	References
In situ growth	Simple process	Poor controllability, random growth	[13,14,24,25,26,28]
Secondary growth	Controllable growth, easy to obtain dense membrane	High demand of seed quality	[29,31]
Layer-by-layer method	Reduced synthesis temperature	Multiple steps	[34]
Microwave-induced thermal deposition	Reduced synthesis time, high efficiency	High cost of heating equipment	[36]
Electrochemical method	Easy to meet the requirements of industrial production	Ag fouling on membrane	[48,49,50,51]

## Data Availability

Not applicable.
